# Significant Global Improvements for Opioid Dependent Patients Receiving 8 Sessions of Flexible Trauma Informed Psychological Therapies Whilst on Long Acting Injectable Buprenorphine: 9 Month Findings

**DOI:** 10.1192/bjo.2024.492

**Published:** 2024-08-01

**Authors:** Jan Melichar, Lucie James, James Tucker, Lewis Jones, Emma Dennie

**Affiliations:** 1 Cardiff and Vale UHB, Cardiff, United Kingdom; 2 Aneurin Bevan UHB, Newport, United Kingdom; 3 University of Bath, Bath, United Kingdom; 4 University of South Wales, Newport, United Kingdom; 5 Cardiff University, Cardiff, United Kingdom

## Abstract

**Aims:**

Opioid dependence is associated with adverse physical health, mental health and social consequences. Daily oral opiate substitutes offer some treatment gains but several negative associations including daily dosage fluctuations, long-term reliance on services and negative impact on ability to work.

Long-acting injectable buprenorphine (LAIB) is a new treatment option, extensively used in Wales since 2020. We have shown the many gains, including increased treatment retention, reduced service reliance, improved patient satisfaction and increased capacity for people to move on in their recoveries, are likely to be due to LAIBs unique combination of allostatic μ-opioid receptor agonism (craving reduction) and sustained κ-receptor antagonism (anxiolysis). However, ~50% experience resurfacing of mental health and/or trauma symptoms on LAIB that impedes recovery. The Buvidal Psychological Support Service, commissioned by Welsh Government, seeks to develop the evidence base for provision of rapidly accessible, tiered psychological support alongside LAIB to address this. Here we present initial 9-month findings.

**Methods:**

Tier 1 of the service offers 8 weekly individual therapy sessions, delivered flexibly over 2–6 months, with an experienced trained therapist focused on psychoeducation, co-production of a trauma and compassioned based formulation, and the development of skills to manage current mental health or trauma symptoms.

Pre- and post-evaluation programme assessed efficacy including: EQ5D-5L, Work and Social Adjustment Scale (WSAS), Clinical Global Impressions (CGI), PRO Severity and Clinical Outcomes in Routine Evaluation –10 (CORE-10).

**Results:**

The service launched in March 2023 with 100 referrals in the first 9 months.

35 patients have completed Tier 1, taking between 2 and 6 months to complete.

Patients who completed Tier 1 showed clinically significant reductions in psychological distress and improvements in global functioning, quality of life and perceived mental health difficulties.

These were statistically significant at p < 0.001 for all measures (EQ5D, ICECAPS, WSAS, CGI, PRO, CORE-10) (28< = n <=34).

**Conclusion:**

Rates of retention in treatment are greater than expected amongst this complex client group and the significant global improvements support the notion that those on LAIB present with increased stability and ability to engage in therapy, and that a tiered flexible approach to therapy can promote psychological safety and engagement and sustained recovery.

We propose that a tiered trauma-focused psychology service is well placed to meet the needs of people on LAIB and should be a core component of LAIB treatment in the UK.

